# Dysregulated Purine Metabolism Contributes to Age-Associated Lower Urinary Tract Dysfunctions

**DOI:** 10.20900/agmr20210018

**Published:** 2021-09-29

**Authors:** Lori A. Birder, Edwin K. Jackson

**Affiliations:** 1 Renal-Electrolyte Division, Department of Medicine, University of Pittsburgh School of Medicine, Pittsburgh PA 15261, USA; 2 Department of Pharmacology and Chemical Biology, University of Pittsburgh School of Medicine, Pittsburgh PA 15261, USA

**Keywords:** purine nucleoside phosphorylase, 8-aminoguanine, lower urinary tract, aging

## Abstract

Lower urinary tract (LUT) dysfunction is common in the older adult. Aging is associated with a number of both storage and voiding problems which are classified into syndromes with overlapping symptoms. Despite the prevalence and consequences of these syndromes, LUT disorders continue to be undertreated as few therapeutic options exist. Here, we propose that dysregulated metabolism of purine nucleotides results in an accumulation of uro-damaging hypoxanthine (a source of reactive oxygen species or ROS), which provides a mechanism for defects in sensory signaling and contractility, culminating in abnormal urodynamic behavior.

## INTRODUCTION

It is well recognized that with age the lower urinary tract (LUT) frequently becomes dysfunctional [1-3]. Although the underlying pathophysiological processes mediating age-related LUT dysfunction are not well understood, multiple bladder components, including the mucosal, muscular, stromal and neural elements, are likely involved. Pathological features arise from: (1) vascular changes leading to ischemia); (2) mucosal alterations characterized by increases in mucosal permeability; (3) decreased bladder sensation; and (4) the inability of the urinary bladder extracellular matrix to exhibit normal compliance during filling and storage; and (5) increased or decreased bladder smooth muscle contractility. These pathological changes interact and converge to produce LUT symptoms and signs including bladder overactivity or underactivity, urgency, nocturia and urinary incontinence during filling/storage [1-4].

Despite the prevalence and impact of LUT disorders (LUTDs) on quality of life in humans, LUTDs continue to be undertreated as few effective therapeutic options exist. Emerging evidence, however, suggests that the enzyme purine nucleoside phosphorylase (PNPase) participates in age-related oxidative injury and damage to cellular structures in the bladder [5-8]. Inhibition of the enzyme PNPase can increase levels of ‘uro-protective’ precursors (e.g., inosine and guanosine) [9-11] and also decreases the levels of ‘uro-toxic’ products (e.g., hypoxanthine and xanthine) [12].

## PURINE METABOLISM: URO-PROTECTIVE ROLE OF INOSINE AND URO-DAMAGING ROLE OF HYPOXANTHINE

PNPase is an enzyme that is expressed in most tissues [13]. This enzyme belongs to the family of glycosyltransferases, is expressed in both bacteria and mammals and is a key enzyme known to be involved in the purine salvage pathway [14,15]. This enzyme is responsible for the transformation of purines inosine and guanosine to their respective bases (i.e., inosine into hypoxanthine and guanosine into guanine) [5,6,13]. Both inosine and guanosine have been shown to exhibit beneficial anti-inflammatory and tissue-protective effects in a wide range of target organ systems including the LUT. In contrast, elevated levels of inosine’s downstream metabolite *hypoxanthine* over time may exhibit harmful effects due to *production of ROS* when metabolized by xanthine oxidase to xanthine and then to uric acid. In addition, hypoxanthine, via the transporters ENT1 and 2 [16,17], can be transported across cell membranes. In this way, urinary hypoxanthine can gain access to underlying tissues within the bladder wall increasing oxidative stress and damaging all components of the lower urinary tract system. There is substantive evidence that oxidative damage by ROS is deleterious to cells and also is involved in the progression of a number of diseases. Because PNPase inhibition blocks the metabolism of inosine to hypoxanthine and guanosine to guanine, and because guanine is metabolized to xanthine by guanase, likely the uro-protective effects of PNPase inhibitors in general, and 8-aminoguanine in particular, are mediated by increases in bladder levels of inosine and guanosine (uro-protective purines) and reductions in bladder levels of hypoxanthine and xanthine (uro-damaging purines). Unlike other treatments such as allopurinol (which only targets hypoxanthine metabolism), blocking PNPase increases “uro-protective” precursors (inosine and guanosine) while simultaneously decreasing levels of “uro-toxic” metabolites (hypoxanthine and xanthine) [7].

## DECREASED “RESILIENCE” IN AGING: IMPACT OF OXIDATIVE STRESS

Studies have shown that changes in both bladder afferent nerve morphology and function with increasing age can impair urinary bladder storage and voiding functions. Age-associated changes in bladder sensation may be due in part to anatomical and functional changes in peripheral nerves as well as altered blood flow that impacts nerve endings [18]. In general, the older adult are much less sensitive to mechanical stimuli and often exhibit reduced ability to detect vibration, touch and pressure [19-21]. Studies in animals confirm that oxidative stress along with increased free radical damage [22] contributes to aging of the peripheral nervous system [23]. Oxidative stress may be produced by bladder ischemia and repeated ischemia/reperfusion during a micturition cycle [24,25]. Complicated changes in both the structure and function of the vasculature are associated with aging and can be associated with a reduction in blood flow and ischemia. Chronic bladder ischemia could lead to neural and urothelial/smooth muscle (SM) damage as well as increased expression of tissue damaging agents.

With advanced age there is likely to be a number of changes including decreased SM cell function and increased fibrosis or stiffening of the bladder (decreased compliance). These and other changes can result in increased urinary urgency and/or inability to empty the bladder well [26,27]. For example, patients with geriatric voiding dysfunction (e.g., impaired contractility) have been shown to exhibit distinctive changes in smooth muscle including cellular atrophy and degeneration [26]. The aged rat detrusor SM exhibits similar structural alterations as reported in older adults, including SM degeneration, swelling and disruption of the mitochondria and abnormalities in proteins associated with mitochondrial health [7]. Because mitochondria are considered the powerhouse of organelles, generating 95% of all cellular energy, these pathological changes in mitochondrial structure and function clearly participate in age-related LUTDs. Mitochondrial decline is known to be one of the key hallmarks of aging and age-related disorders [28,29].

In addition, age-related changes in the extracellular matrix (ECM) may also impact the function of various cell types within the bladder wall [25]. Despite having different etiologies, many types of chronic fibrotic disorders are associated with increased oxidative stress which, over time, progressively destroys the organ’s architecture and in turn its function [30,31]. During normal bladder filling, the coordinated recruitment of collagen fibers across the bladder wall (both SM and lamina propria), is lost in the aged bladder [32]. This loss of bladder elasticity impacts the ability of the urothelium (UT) to sense mechanical changes that take place during a micturition cycle and, in turn, release mediators that influence sensation. For example, urothelial release of ATP, acting on purinergic receptors which reside on subepithelial sensory nerve terminals, are a key part of a primary mechanical sensing system in the bladder [33].

## SUMMARY

It is clear that: (1) age-related LUTDs are prevalent and increasingly so because of the aging of a substantial segment of most societies; and (2) there is an unmet need for effective and safe treatments for LUTDs. Because ROS (produced by multiple pathways) are involved in the pathophysiology of aging-related bladder dysfunction, age-related bladder dysfunction does indeed fall into the category of a complex disease that will require pleiotropic drugs to manage. In this regard, and as mentioned above, hypoxanthine gives rise to ROS via its downstream metabolism by xanthine oxidase. There is emerging evidence that changes in the activity of PNPase may lead to an abnormal urinary bladder purine metabolome, which in turn causes bladder and urethral inflammation, oxidative injury, and cellular damage [7,8]. Therefore, inhibition of PNPase could be used to manipulate the urinary bladder purine metabolome- by simultaneously increasing uro-protective and decreasing uro-damaging purine metabolites. Thus, PNPase inhibitors may prevent age-associated deterioration of the LUT and decrease the burden of LUT diseases.
